# Identification of a Novel Gene Signature of ES Cells Self-Renewal Fluctuation through System-Wide Analysis

**DOI:** 10.1371/journal.pone.0083235

**Published:** 2014-01-02

**Authors:** Luigi Cerulo, Daniela Tagliaferri, Pina Marotta, Pietro Zoppoli, Filomena Russo, Claudia Mazio, Mario DeFelice, Michele Ceccarelli, Geppino Falco

**Affiliations:** 1 Department of Stem Cell and Development, Istituto di Ricerche Genetiche Gaetano Salvatore Biogem scarl, Ariano Irpino, Italy; 2 Department of Science, Università degli Studi del Sannio, Benevento, Italy; 3 Department of Medicina Molecolare e Biotecnologie mediche, Università di Napoli Federico II, Naples, Italy; Wellcome Trust Centre for Stem Cell Research, United Kingdom

## Abstract

Embryonic Stem cells (ESCs) can be differentiated into ectoderm, endoderm, and mesoderm derivatives, producing the majority of cell types. In regular culture conditions, ESCs' self-renewal is maintained through molecules that inhibit spontaneous differentiation enabling long-term cellular expansion. This undifferentiating condition is characterized by multiple metastable states that fluctuate between self-renewal and differentiation balance. Here, we aim to characterize the high-pluripotent ESC metastate marked by the expression of *Zscan4* through a supervised machine learning framework based on an ensemble of support vector machine (SVM) classifiers. Our study revealed a leukaemia inhibitor factor (Lif) dependent not-canonical pluripotency signature (*AF067063*, *BC061212*, *Dub1*, *Eif1a, Gm12794*, *Gm13871, Gm4340*, *Gm4850*, *Tcstv1*/3, and *Zfp352*), that specifically marks *Zscan4* ESCs' fluctuation. This novel ESC metastate is enhanced by high-pluripotency culture conditions obtained through Extracellular signal Regulated-Kinase (ERK) and Glycogen synthase kinase-3 (Gsk-3) signaling inhibition (2i). Significantly, we reported that the conditional ablation of the novel ESC metastate marked by the expression of *Gm12794* is required for ESCs self-renewal maintenance. In conclusion, we extend the comprehension of ESCs biology through the identification of a novel molecular signature associated to pluripotency programming.

## Introduction

Embryonic stem cells (ESCs) are derived from the inner cell mass of blastocyst and are characterized by two remarkable peculiarities, namely self-renewal and pluripotency: self-renewal is defined as the symmetrical division of ESCs into identical undifferentiated daughter cells; pluripotency confers to ESCs the ability to produce the majority of cell types. It has become evident over the past few years that ESCs` within the same culture condition fluctuate among different levels of potency [1], [2], [3] as consequence of paracrine effects and cell-to-cell interactions that are not homogeneously regulated with current *in vitro* culture conditions. Consistently, ESC mosaic-in colony expressions of key canonical pluripotency genes such as *Nanog* and *Rex1* (reduced expression protein 1) reflect the temporal heterogeneous expression at single cell level profoundly affecting the state of pluripotency [4], [5]. Recently, a novel transient ESCs state (metastate) was reported, referred as a high level of pluripotency [6], characterized by the remarkable potential to produce both embryonic and extra-embryonic cell lineages [7]. This metastate is observed in a small fraction of the ESCs population, and it is marked by the expression of *Zscan4* (zinc finger and SCAN domain containing 4), a key factor required for ESC genome stability and to increase the reprogramming efficiency of induced pluripotent stem (iPS) cells [6], [8]. The comprehension of the gene network underlying such ESCs metastate represents a suitable opportunity to understand the pluripotency maintenance and to enhance applications in tissue regeneration [9], [10], [11], [12], [13]. Significant steps have been made towards the molecular characterization of high pluripotent ESC metastate through the analysis of multiple global gene expression profiles, yielding an extensive list of putative candidates [3], [7]. However, beyond *Zscan4* the genes that are functionally relevant to a high pluripotency metastate is still a matter of debate. In the present work, we aim to identify genes that are involved in the maintenance of the high pluripotency ESCs metastate marked by *Zscan4*. In particular, we developed a supervised machine-learning framework to predict the genes that are functionally related to the *Zscan4* mechanism in ESCs. The supervised machine learning framework was based on an ensemble of support vector machine (SVM) classifiers [14], [15], trained with the expression of a small cohort of genes, which have been related to *Zscan4* over several ESC experimental conditions [3], [6], [7].

The molecular characterization of gene hypotheses predicted by our supervised machine learning framework revealed a novel high pluripotency gene signature (*AF067063*, *BC061212*, *Dub1*, *Eif1a, Gm12794, Gm13871, Gm4340*, *Gm4850*, *Tcstv1*/3, and *Zfp352*), that enabled the identification of different *Zscan4* metastate populations. Moreover, we functionally proved, by cell ablation, that the Zscan4 subpopulation marked by *Gm12794* is required for ESCs pluripotency maintenance suggesting the existence of different levels of high pluripotency. Our study extends the comprehension of ESCs biology through the identification of a novel molecular network associated to pluripotency programming.

## Materials and Methods

### Dataset selection

We collected a set of deposited ESCs DNA microarray datasets in which the expression of at least one SEED (genes *AF067063*, *BC061212*, *Eif1a*, *Gm12794*, *Gm4340*, *Pif1*, *Tcstv1*/3, and *Zscan4*) member was perturbed (Table S1) [16], [17], [18], [19], [20], [21], [22]. To make each experimental condition homogeneous, quantile normalization was applied to the whole dataset to overcome inter-experiment variability as CEL files were not available for all experiments [23]. Probes with low inter experiment variability were filtered out by means of the varFilter function of the genefilter Bioconductor package. Such a function estimates the interquartile range (IQR) for each probe excluding those with an IQR less than a used defined threshold. We adopted the maximum threshold that does not exclude any of the SEED members. This reduced the original set of 45101 probes to 29577 probes over a total of 56 different conditions. The data were finally scaled to zero mean and unit standard deviation.

### Mapping to UCSC genes

To compare different microarray platforms each probe was mapped to the corresponding UCSC gene ID. For the Agilent platform we adopted the blat UCSC tool with a tolerance of 57 nucleotides matches out of 60 nucleotides, which is the length of Agilent probes, to retrieve the locations of UCSC gene on the mm9 assembly. For the Affymetrix MOE 430v2 platform we adopted the annotation information provided by Netaffx that maps each affy probe on the genome mm9 assembly coordinates.

### Clustering Gene families

To detect gene families we clustered together genes having a short blast transcript sequence distance. To this aim we adopted the blast distance pre-computed by UCSC and available through the file knownBlastTab.txt, and the hierarchical clustering algorithm configured with Euclidean distance and Ward linkage. A cut level of 90% was adopted to determine the set of cluster families.

### Retroelement (LTR, SINE/Alu), GC, and H3K9me2 analysis

We determined the overall coverage by retroelements (LTRs and SINE/Alus) in a 2.5 kb window (2.0 kb upstream and 0.5 kb downstream) surrounding the 5′ terminus of the annotated transcripts. Annotations generated by RepeatMasker (v3.3.0) from the Dec 2011 assembly of the mouse genome (GRCm38/mm10) were used to obtain the attributes for all repeat elements. The overall LTR and SINE/Alu coverage surrounding the 5′-terminus of MGS transcripts was compared to 5000 randomly selected sets of genes with the same number of genes obtaining a significant enrichment. For GC percentage levels we extracted a 2.0 kb window (1.0 kb upstream and 1.0 kb downstream) surrounding the 5′-terminus of the annotated transcripts from the December 2011 assembly of the mouse genome (GRCm38/mm10) and computed the GC level with the UCSC faCount tool. We adopted a t-test to evaluate whether the level of GC is significantly higher in the promoter region of MSG genes.

### Culture of ESCs

The mouse ESCs line E14Tg2a.4 derived from strain 129P2/OlaHsd [24], were cultured for two passages on gelatin-coated feeder-free plates and subsequently maintained in gelatin-coated six-well plates in complete ES medium: GMEM (Glasgow Minimum Essential Medium, Gibco), 15% FBS (EuroClone), 1,000 U ml-1 leukaemia inhibitory factor (LIF) (EuroClone), 1.0 mM sodium pyruvate (Invitrogen), 0.1 mM non-essential amino acids (Invitrogen), 2.0 mM L-glutamine (Invitrogen), 0.1 mM β-mercaptoethanol and 500 U ml-1 penicillin/streptomycin (Invitrogen). The cells were incubated at 37°C in 6% CO_2_; medium was changed daily and cells were split every 2 to 3 days routinely.

### Primer design

PCR primer pairs were designed with the Vector NTI software (Invitrogen, Carlsbad, CA, USA) and were tested using ESCs' cDNA with SYBR Green PCR Master Mix (Applied Biosystems, Foster City, CA, USA). First, each primer pair was run using a matrix of forward and reverse primers with various concentrations, and threshold cycle measurements were compared with dissociation curves to determine optimal primer concentrations with high amplicon specificity. Second, a 5-log standard curve dilution series was run using each primer pair at the optimal concentration, and amplification efficiencies were calculated. Primer sets with suboptimal dissociation curves or amplification efficiencies outside of the 85–115% range were discarded.

### Quantitative Real-Time Polymerase Chain Reaction (qRT-PCR)

One microgram of total RNA, isolated from cells by TRIzol (Invitrogen), was reverse-transcribed by Quantitec reverse transcription kit (Qiagen) according to the manufacturer's instructions. qPCR analyses were performed using 7.5 ng cDNA per well in duplicate with the SYBR green master mix (Applied Biosystems) according to the manufacturer's instructions. Reactions were run on 7900HT system (Applied Biosystems). Fold induction was calculated and normalized by the ΔΔCt method.

### RNA *In situ* hybridization

Cells were fixed in 4% PFA/PBS at 4°C overnight. After digestion with proteinase K, cells were hybridized overnight with 1 µg digoxigenin-labeled riboprobe or fluorescein-labeled riboprobe at 60°C. Cells were then washed, blocked, incubated with alkaline phosphatase-conjugated anti digoxigenin antibody and incubated with NBT/BCIP detection buffer for 30 min. For double *In situ* hybridization cells were incubated with anti digoxigenin antibody (1∶2000; Roche) and anti fluorescein antibody (1∶500; Abcam). To prepare RNA probe preparation, 200 ng of cDNA were PCR-amplified in 50 µl PCRs using SP6 (5′-GATTTAGGTGACACTATA-3′) and T7 (5′-TAATACGACTCACTATAGGGA-3′) primers. PCR products were purified using a QIAquick PCR purification Kit (Qiagen), eluted in 30 µl of buffer, and quantitated using a NanoDrop. Digoxigenin-labeled RNA probes were transcribed from the PCR product templates using DIG RNA Labeling Kit (Roche) and the appropriate RNA polymerase. Probes were purified through RNA column and quantitated by agarose gel electrophoresis or by running an RNA 6000 Nano Assay on a 2100 Bioanalyzer.

### RNA *In Situ Hybridization* and Immunofluorescence Staining

ESCs were plated on gelatin-coated feeder-free plates. Cells were fixed with 4% paraformaldhehyde (PFA) for 30 min, followed by washing with PBS-T (0.05% tween). Cells were hybridized overnight with 1 µg digoxigenin-labeled riboprobe at 60°C. Cells were blocked with Blocking Solution (Roche) and stained with primary antibodies for 1 h at room temperature. Antibodies used: sheep anti digoxigenin antibody (1∶2,000; Roche), rabbit anti OCT3/4 (1∶500; Abcam) and rabbit anti NANOG (1∶500; Abcam). After washing three times for 5 min with PBS-T, cells were stained with secondary antibodies (1∶200 anti sheep and rabbit IgG Alexa fluor 488 and 594) for 30 min at room temperature and washed again three times with PBS-T. Cells were stained with DAPI in PBS for 2 min and then imaged using a fluorescence microscope and oil objective.

### Cell Ablation Strategy

To generate the pGm12794-Strawberry vector, the Strawberry gene was amplified with the couple of primers *KpnI*-*AscI*-*EcoRV*-StrawF1 (5′-atggtgagcaagggcgaggagaataac-3′) and *BglII*-StrawR1 (5′-ctacttgtacagctcgtccatgccg-3′); the bgHpA poly(A) signal was amplified from the plasmid pL452 (from National Cancer Institute – Frederick) using the couple of primers *BglII*-pAf (5′-cttcttgacgagttcttctgagggg-3′) and *EcoRI*-*SalI*-pAr (5′-gttatattaagggttccgcaagc-3′). Both PCR products were cloned in pL452 using the *KpnI*-*BglII*-*EcoRI* restriction sites. Finally, a 5.0 kb region, upstream the ATG of the *Gm12794*, was amplified from the BAC bMQ299i11by PCR using the primers pRNIf (5′-ttcaaaggctgctagtggaagactg-3′) and pRNIr (5′-ataatttcaggctaagttttggaaattcc-3′) and was inserted in pCR-XL-TOPO (Invitrogen), from which was cut *MluI*-*EcoRV* and ligated in pL452-Strawberry digested *AscI*-*EcoRV*. To generate the pGm12794-TK vector, a DNA fragment containing the thymidine kinase (TK) coding sequence completed of a poly(A) signal was amplified with the couple of primers *HpaI*-TKf (5′-agcgcgtatggcttcgtacc-3′) and *SalI*-TKr (5′-cttgataccccacgcaacgc-3′). This fragment was inserted in the pGm12794-Strawberry digested *EcoRV*-*SalI*, replacing the Strawberry-pA sequence. All the passages of the plasmids construction were verified by sequence analysis.

## Results

### A supervised machine learning framework reveals a Main Gene Signature (MGS) hypothesis

**Figure 1:**
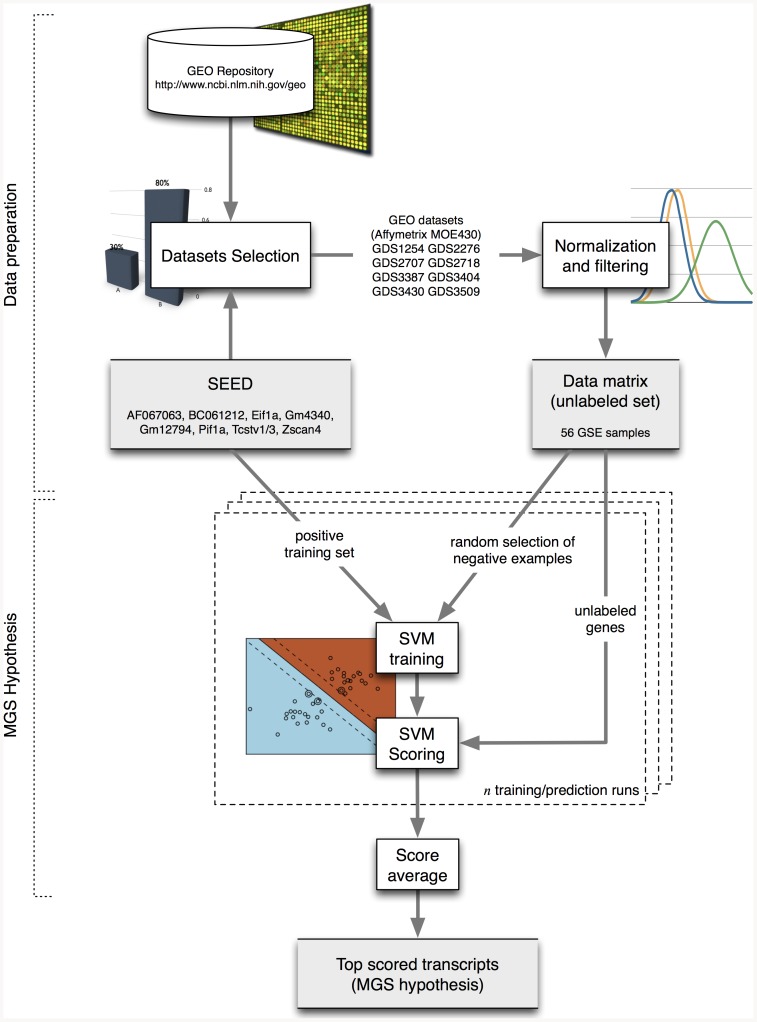
MGS Hypothesis. SEED genes are used to guide the selection of 8 GEO datasets. Those experiments are merged, and normalized, in order to obtain a single dataset consisting of 45101 Affymetrix 430 2 probes and 56 GSE experimental conditions. SEED genes are the our positive training examples in a positive-only Support Vector Machine (SVM) classification framework composed of 1000 classifiers. To overcome the lack of counterpart members each classifier is trained with a random subsets of genes adopted as negative examples, leaving the SEED genes fixed as positive examples. At each training/prediction run the remaining unlabeled genes are scored according to the classifier. The Main Gene Signature (MGS) hypothesis is obtained considering the top 100 genes ranked by averaging the scores among all random SVM classifiers.

**Figure 2:**
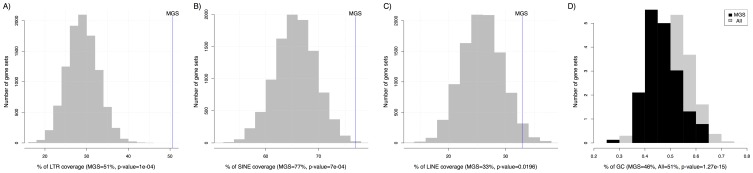
MGS In-silico evaluation. The overall coverage of retroelements detected with RepeatMasker (v3.3.0) was determined in a 2.5 kb window (2.0 kb upstream and 0.5 kb downstream) surrounding the 5′ terminus of the mouse genome (GRCm38/mm10) annotated transcripts. The GC percentage levels in a 2.0 kb window (1.0 kb upstream and 1.0 kb downstream) surrounding the 5′-terminus of the mouse genome (GRCm38/mm10) annotated transcripts were determined with UCSC faCount tool. The x-axes show the coverage level of retroelements or GC percentage, while the y-axes show the number of gene sets that exhibit that coverage level. The distribution of the coverage levels of 5000 randomly selected sets of genes with the same number of MGS genes is showed in grey respectively for: A) LTR (average level of random distribution 34%); B) SINE/Alu (average level of random distribution 67%); C) LINE (average level of random distribution 26%); D) GC% (average level of random distribution 51%). The vertical lines show the coverage levels of the MGS set. A significant enrichment is observed for LTR and SINE retroelements (p-value<0.001); t-test shows that the level of GC% in the promoter region of MGS genes is significantly lower (p-value = 1.27 10^−15^).

In order to identify an accurate Main Gene Signature (MGS) functionally related to the ESC high-pluripotency level marked by *Zscan4*, we implemented a supervised machine learning framework based on an ensemble of Support Vector Machine (SVM) classifiers [25], [26]. Besides *de novo* approaches, such as clustering based on expression correlation, supervised algorithms are able to predict novel non linear gene functional relationships on the basis of known examples (called training examples). The overall procedure is sketched in Figure 1. We considered the expression profiles of *AF067063*, *BC061212*, *Eif1a*, *Gm4340*, *Gm12794*, *Pif1a*, *Tcstv1*/3, as a reference model to train SVM classifiers, as this set of genes is known to be correlated with the high-pluripotency metastate marked by *Zscan4* [3]. Hereafter, we will refer to this cohort of genes, including *Zscan4*, as SEED. We assembled 8 GEO datasets (GDS), comprising a total of 56 global gene expression profiles (GSE), corresponding to different ESC experimental conditions in which the expression of at least one SEED member was perturbed (Table S1). The MGS prediction was performed by training 1000 SVM classifiers having the SEED genes fixed as positive examples, and a random subsets of genes as negative examples. The trained classifiers were then used to score all the genes. Afterwards, the final score of each unlabelled gene was obtained by averaging the classification score obtained by that gene in all the SVM runs. Finally, in accordance to their ranking score we considered the top 100 genes as the MGS hypothesis (Table S2) corresponding to 52 annotated genes (because 70 transcripts shared a nucleotide identity above 90% and were clustered in 22 annotated genes (Table S2)). Noticeably, in MGS list there is *Dux4* (double homeobox 4), a gene that was recently shown to directly bind the promoter of *Zscan4*, and *Dppa3* (development pluripotency associated 3), a marker of ESC pluripotency metastate, whose expression pattern is also known to be ESC mosaic-in-colony [9]. Consistently with the gene enriched in the high pluripotency metastate [7], we found that also the upstream region to the transcriptional start sites of MGS genes were significantly enriched by LTR retroelements insertions (LTR and SINE, p-value≤0.001, Fig. 2A and 2B), and presented a level of GC percentage significantly low (46%, *p*-value = 1.27 10^−15^, Fig. 2D) with respect to all mouse genes. Altogether, these data strongly supported the hypothesis that MGS is a putative molecular signature underlining the ESC high level of pluripotency marked by *Zscan4*.

### A novel cohort of ESC mosaic-in-colony cell markers

**Figure 3:**
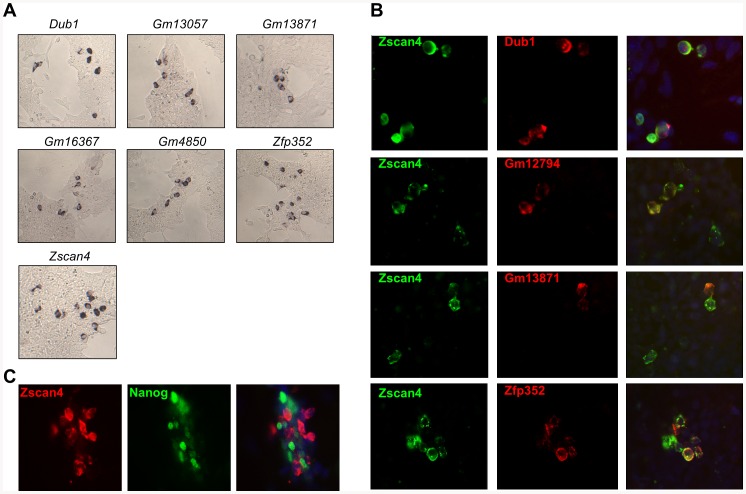
ESCs heterogeneous subpopulation. Gene expression pattern in ESCs cultures by *in situ* hybridization after 3 days in culture. A) RNAs are detected by a BCIP/NBT system producing a deep purple reaction product. RNA *ISH* representative “spotted” patterns on ESCs colonies (20X). The panel B shows double RNA *ISH* stain (*red*) (*Dub1, Gm12794, Gm13871*, and *Zfp352*), and Zscan4 RNA ISH staining (*green*), counterstained with DAPI (*blue*) (63x). The panel C shows double stain through *Zscan4* RNA ISH (red) and NANOG immune-staining (green), counterstained with DAPI (blue) (63x).

Considering that the high pluripotency metastate is shared by a subset of ESC cells, we focused our attention on genes whose expression manifested ESC mosaic-in-colony patterns. To evaluate our hypothesis, we characterized the ESC spatial expression of each member of the MGS by RNA *in situ* hybridization (ISH) methodology. We showed that in addition to *Dppa3*, the MGS included also 6 novel ESC mosaic-in-colony expressed genes: *Dub1*, *Gm13057*, *Gm13871*, *Gm16367*, *Gm4850*, and *Zfp352* (Fig. 3A). *Dub1* (deubiquinating enzyme 1) is significantly expressed during the ESCs high pluripotency metastate [7]; *Gm4850* and *Gm13057* are predicted genes with high conservation to *Tho4* complex [27], and *Pramel* families respectively [28]; *Gm13871* has no known domain nor a known function; *Gm16367* has a conserved protein domain involved in the nuclear export of pre-ribosomes; *Zfp352*, a zinc finger protein significantly expressed during mouse preimplantation development [29], has unknown function. In accordance with our hypothesis we considered the genes having the mosaic-in-colony pattern as potential markers of the high-pluripotency metastate.

### Identification of a novel ESC high pluripotency gene signature

**Figure 4:**
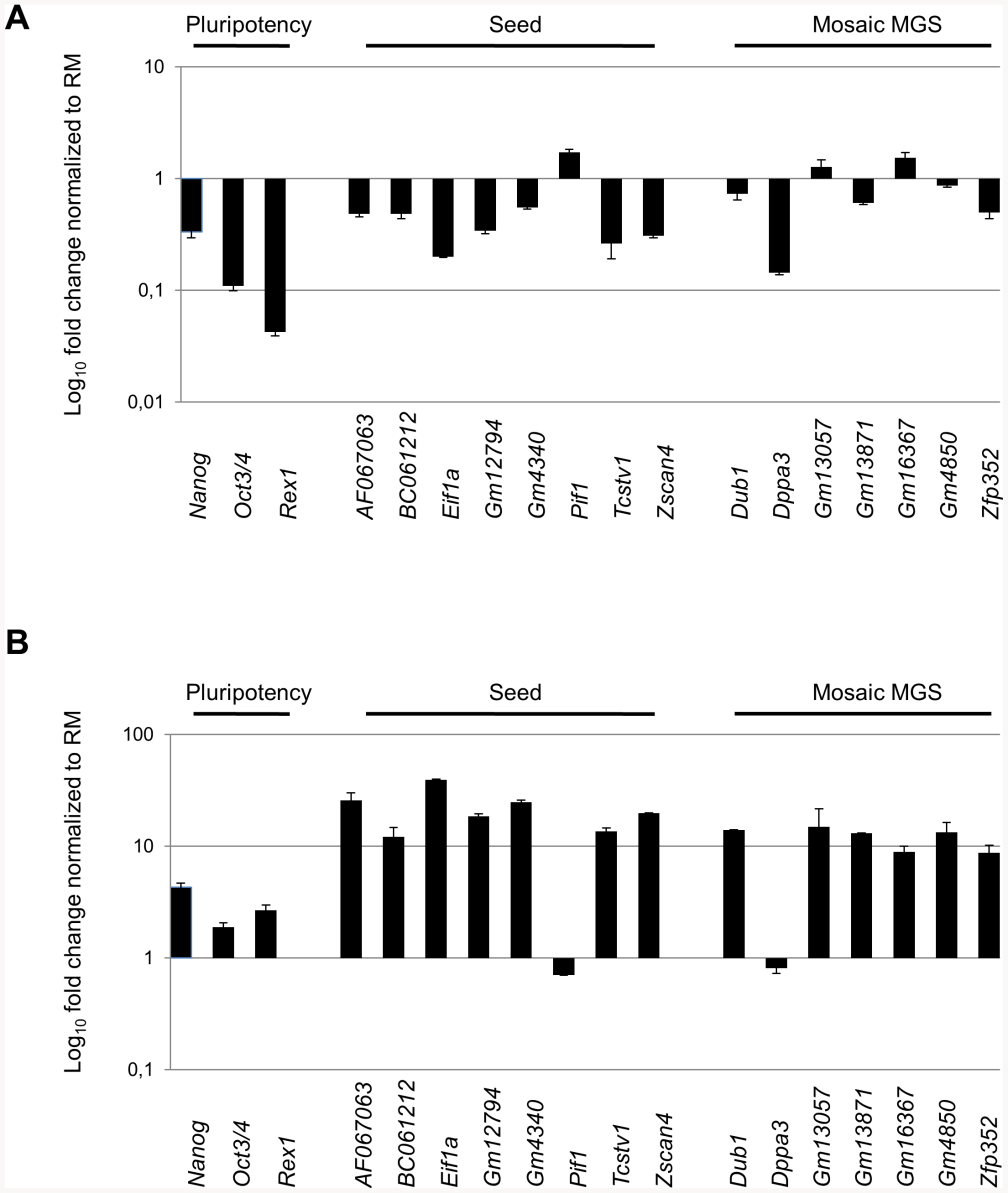
SEED and mosaic MGS expressions in ESCs. ESCs are cultured for 5 days in: *RM*, *Lif-*, and *RM* supplemented with PD0325901 (0.5 µM, Stemgen), and CHIR99021 (3.0 µM, Stemgen) which prevent differentiation by inhibiting ERK, and Gsk3 (*2i*). The mRNA expression levels are assessed by *Real Time* PCR and normalized by Regular Medium (RM) condition in *Lif-* (*panel A*) and in ground state condition (*panel B*). The ESC differentiation condition and the ESC *ground state* condition are evaluated through the expression levels of key canonical markers of pluripotency such as *Oct3/4, Nanog*, and *Rex1* that we considered as positive controls.

We evaluated whether the expressions of MGS mosaic-in colony genes and SEED members were modulated in ESC culture conditions affecting levels of pluripotency. The low-pluripotency condition was obtained by withdrawing *Leukemia Inhibitory Factor* (hereafter *Lif-*), a key cytokine for the maintenance of pluripotency, from the regular ESC defined medium. The high-pluripotency condition, defined ESC *ground state*, was obtained by supplementing the ESCs defined medium with the cytokine *Lif* and two inhibitors (*2i*) (which together block extracellular signal-regulated kinases [ERKs] cascade and glycogen synthase kinase 3b (GSK3b)) [30]. We found that *AF067063*, *BC061212*, *Dppa3*, *Eif1a, Gm12794, Gm13871, Gm4340, Tcstv1*/3, *Zfp352* together with *Zscan4* were strongly downregulated in the *Lif-* condition; *Dub1* and *Gm4850* were barely downregulated; *Pif1*, *Gm13057*, and *Gm16367* were upregulated (Fig. 4A). We found that all candidates, with the exception of *Pif1* and *Dppa3*, were significantly upregulated (more than 10 folds) in the *ground state* pluripotency condition (Fig. 4B). Altogether, *AF067063*, *BC061212, Dub1, Eif1a*, *Gm12794, Gm13871, Gm4340, Gm4850, Tcstv1*/3, and *Zfp352* were positively associated with the Lif dependent high-pluripotency culture conditions.

### Zscan4 positive ESCs consist of multiple subpopulations

We investigated whether the expression between *Zscan4* and each high pluripotency novel candidates was associated through double fluorescent ISH. Interestingly, we found that *Dub1*, *Gm12794, Gm13871* and *Zfp352*, stained small fractions of the ESC *Zscan4* subpopulation (Fig. 3B). This data indicated that ESCs high pluripotency level marked by *Zscan4* expression is not a homogeneous population but consists of multiple subpopulations. We were not able to produce double staining *Gm13057*, *Gm16367* and *Gm4850*, because the fluorescent ISH riboprobes were less sensitive than the chromogenic ISH riboprobes.

Since it is known that *Nanog* expression marks a transient high level of pluripotency [5], we further investigated the relationship between NANOG and *Zscan4* subpopulations. ESCs were immunostained with NANOG antibodies, and hybridized by fluorescent RNA ISH with *Zscan4*. Noticeably, the double staining data revealed no overlap between NANOG signals and *Zscan4* positive cells (Fig. 3C). Altogether our data indicated that ESC cultures consist of multiple but not overlapping levels of pluripotency (Fig. S1).

### *Gm12794* is required for ESCs self-renewal maintenance

Among multiple pluripotency subpopulations, we aimed to characterize the role of the ESC marked by the expression of *Gm12794* because it belongs to the Prame family, which it has been closely associated to balance the undifferentiated and the differentiated states of ESCs [28] thus resulting in a potential metastate marker. We investigate the relationship between *Gm1279* cells and ESC pluripotency by selective ablation of *Gm12794* expressing cells (Gm12794^+^) using herpes simplex 1 virus thymidine kinase (HSVTK) system. HSVTK expression in transgenic ESCs is not toxic but it renders cells sensitive to the nucleoside analog ganciclovir (GCV), allowing targeting cell ablation.

**Figure 5:**
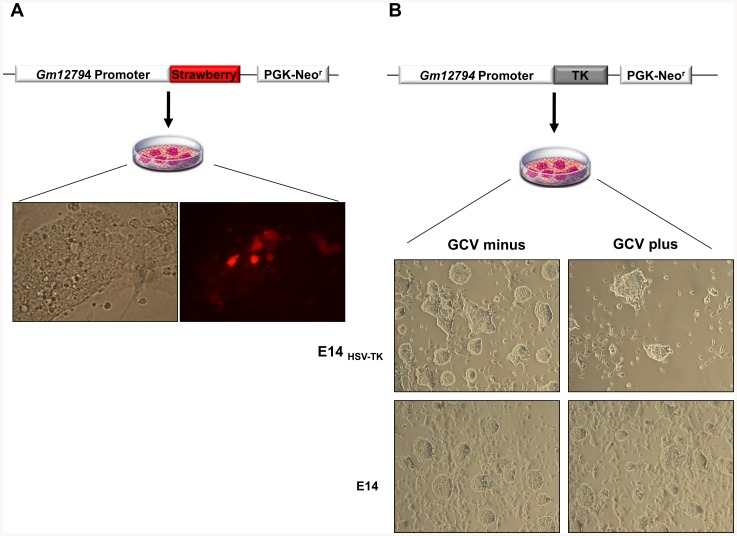
Ablation of Gm12794 expressing ESCs. A) Schematic diagram of the p*Gm12794*-Strawberry reporter vector. The reporter gene *Strawberry* is placed under the control of a 5.0 kb region upstream the *Gm12794* ATG start codon. A PGK-Neo^r^ cassette is used for the selection of the ESC clones; the *Gm12794*-Strawberry reporter electroporated in mESCs is visualized by the Strawberry reporter gene (20x). B) Schematic diagram of the *Gm12794*-HSV-Tk vector electroporated in mESCs to generate ESC*^Gm12794_HSVTK^* lines. ESC*^Gm12794_HSVTK^* lines and control ESC*^HSVTK^* lines were cultured in presence or absence of Ganciclovir (GCV) (2.0 µM, Sigma) (20x).

First, we demonstrated that 5.0 kb of 5′ flanking sequence of the *Gm12794* was sufficient to drive gene expression by generating Strawberry reporter transgenic ESC line (Fig. 5A). The Strawberry signals marked ESC mosaic-in colony subpopulation, and it was consistent with the existence of Gm12794^+^ ESC subpopulation.

Second, to target an inducible toxic phenotype of Gm12794^+^, we generated the ESC transgenic line in which the expression of HSVTK was under the control of the identified *Gm12794* promoter (Fig. 5B). The ESC*^Gm12794_HSVTK^* were cultured in media with GCV, and without GCV, and were analysed. The ESC^5*Gm12794_HSVTK*^ cultured in presence of GCV formed fewer colonies than ESC*^Gm12794_HSVTK^* cultured without GCV (GCV*^plus^* = 26±5; GCV*^minus^* = 63±6 colonies; number of replicates = 4). The parental ESC line was barely affected by the addition of Ganciclovir (GCV*^plus^* = 62±4; GCV*^minus^* = 68±5 colonies; number of replicates = 3). These data indicated that Gm12794^+^ cells represent a transient ESC subpopulation metastate required for the maintenance of ESCs.

## Discussion

The gene *Zscan4*, a critical factor for chromosomal stability, is expressed heterogeneously in the conventional culture of ESCs [4], [5]. It has become evident that Zscan4 ESC expression heterogeneity reflects a stochastic transition marking an ESC high pluripotency metastate [7], [8]. To comprehend the molecular network underlining the Zscan4 ESC metastate, we implemented a system-wide bioinformatics analysis of the transcriptome of a wide set of ESC experiments generating the *Zscan4* metastate Main Gene Signature (MGS) hypothesis. The experimental validation of the MGS hypothesis revealed that the supervised learning approach was successful in improving the molecular characterization and functional relationships of the *Zscan4* ESC metastate. In particular, our study revealed that the *Zscan4* ESC metastate consist of multiple molecular levels, revealed by the expression of: *AF067063*, *BC061212, Dub1, Eif1a*, *Gm12794, Gm13871, Gm4340, Gm4850, Tcstv1*/3, *Zfp352*. In particular, we functionally characterized the ESCs metastate marked by *Gm12794*, a novel member of the Prame family, demonstrating that is required for self-renewal maintenance. Noticeably, although these novel ESC metastates were significantly enhanced in the pluripotent *naïve ground state*, they do not express *Nanog*. The co-existence of canonical and not-canonical levels of ESC pluripotency in defined culture conditions could explain why *Nanog* is disposable for ESCs pluripotency retention. Recently, two reports [7], [8] independently described and characterized an ESC high pluripotency metastate that are basically the same from the point of view of their gene expression profile, but differ on the basis of their developmental potency. An accurate molecular comparison between the two reported ESC subpopulations revealed that they could represent different *Zscan4* metastate levels and therefore explains the reason of this apparent ambiguity.

In conclusion, this research provides insights to extend the molecular characterization of ESCs biology, reducing ambiguous phenotypes of ESCs progenies. In our opinion, further investigations on the role of *Zscan4* ESC metastates could improve the understanding of the signalling pathways and gene expression regulation fundamental for potential applications such as tissue regeneration and cell replacement.

## Supporting Information

Figure S1 **The signature expression is mutually exclusive of NANOG positive cells.** Double stain through *RNA ISH* (*red*) (*Dub1, Eif1a and Tcstv1/3*), and NANOG immune-staining (*green*), counterstained with DAPI (*blue*) (63×).
(TIF)

Table S1 **Microarray DataSets selection.** In each DataSet chosen, at least one of the seven signature genes is differentially expressed. All the microarray experiments are performed on Affymetrix Gene Chip 430 2.0. According to the sample used for the microarray experiments the DataSets are selected based on their specificity to ESCs manipulation.
(TIFF)

Table S2 **List of MGS prediction.** The MGS hypothesis generated by SVM classification listed following the ranking score. The UCSC identifier is obtained scoring the Affy probes. UCSC transcripts with a nucleotide identity above 90% are numbered in clusters.
(PDF)
