# Supplementary figures and images for: Identification of a Novel Gene Signature of ES Cells Self-Renewal Fluctuation through System-Wide Analysis
Source: PLoS One. 2014 Jan 2;9(1):e83235. doi: 10.1371/journal.pone.0083235 (PMC3879232; doi:10.1371/journal.pone.0083235)

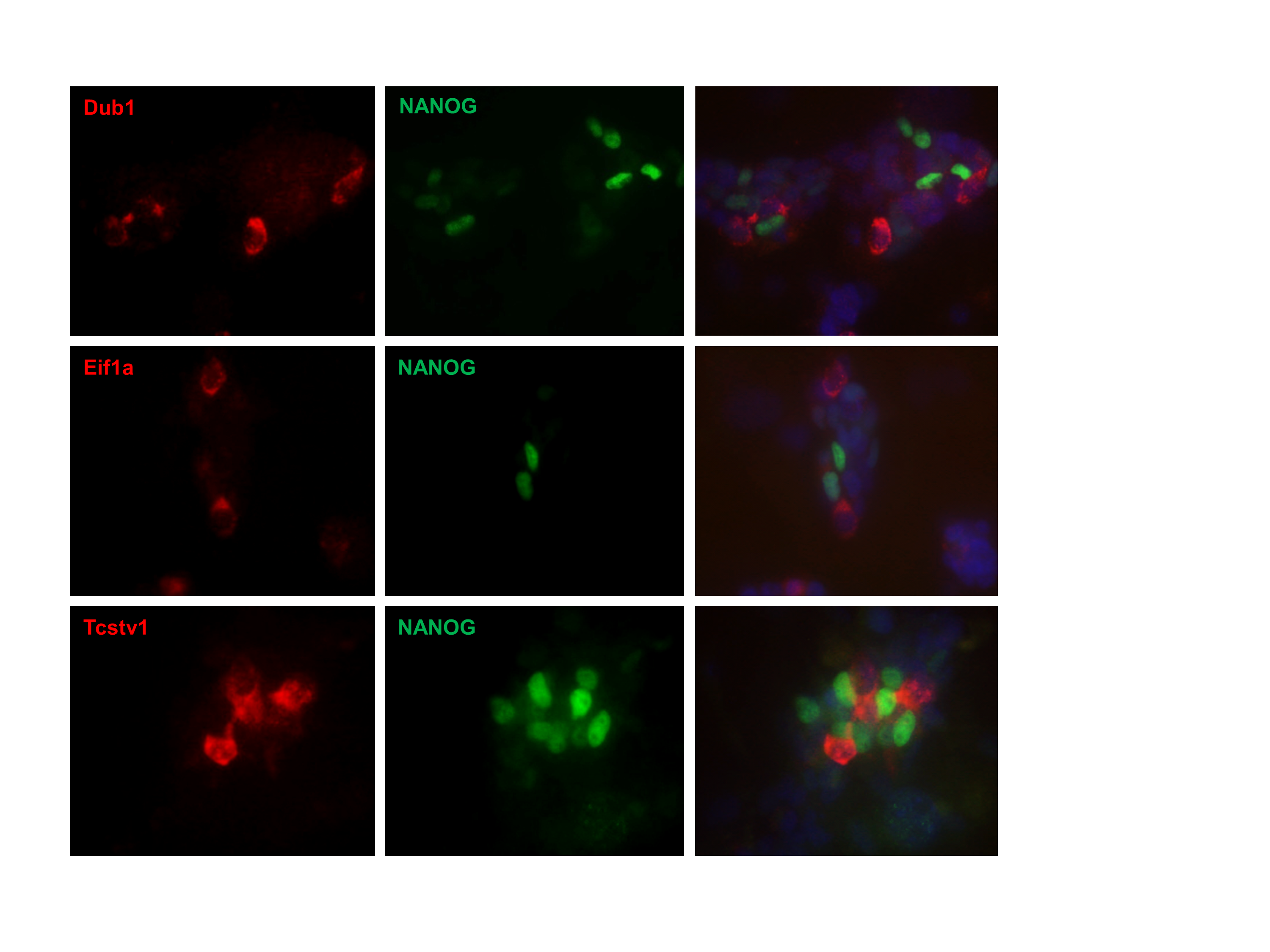

Supplement: Figure S1 — The signature expression is mutually exclusive of NANOG positive cells. Double stain through RNA ISH (red) (Dub1, Eif1a and Tcstv1/3), and NANOG immune-staining (green), counterstained with DAPI (blue) (63×). (TIF) [file pone.0083235.s001.tif]
