# Supplementary material for: Identification of a Novel Gene Signature of ES Cells Self-Renewal Fluctuation through System-Wide Analysis
Source: PLoS One. 2014 Jan 2;9(1):e83235. doi: 10.1371/journal.pone.0083235 (PMC3879232; doi:10.1371/journal.pone.0083235)
Supplement: Table S2 — List of MGS prediction. The MGS hypothesis generated by SVM classification listed following the ranking score. The UCSC identifier is obtained scoring the Affy probes. UCSC transcripts with a nucleotide identity above 90% are numbered in clusters. (PDF) [file pone.0083235.s003.pdf]

UCSC identifier	MGS rank	Gene symbol	Cluster ID
uc008tom.1	1	zfp352	no cluster
uc009qag.1	2	Spesp1	no cluster
uc009fqf.1	3	Tex101	1
uc009fqg.1	4	Tex101	1
uc009fqh.1	5	Tex101	1
uc009fqi.1	6	Tex101	1
uc012ffo.1	7	Tex101	1
uc009sjq.2	8	gm16367	2
uc009sjs.2	9	gm16367	2
uc012ehp.1	10	gm16367	2
uc012ehq.1	11	gm16367	2
uc012hdp.1	12	gm16367	2
uc012hdq.1	13	gm16367	2
uc012hdr.1	14	gm16367	2
uc009iwz.2	15	Dub1	no cluster
uc008tio.1	16	gm13871	3
uc008tiu.1	17	gm13871	3
uc008tiz.2	18	gm13871	3
uc008tja.2	19	gm13871	3
uc008tjb.2	20	gm13871	3
uc008tjc.2	21	gm13871	3
uc008tjd.2	22	gm13871	3
uc008tje.2	23	gm13871	3
uc012dgg.1	24	gm13871	3
uc012hgh.1	25	A2ANE6	no cluster
uc009dpn.1	26	Dppa3	no cluster
uc012hgg.1	27	Rhox3g	no cluster
uc007fcv.2	28	Dux4	no cluster
uc007fcw.1	29	EG245263	no cluster
uc009tfj.2	30	1700013H16Rik	no cluster
uc008koa.1	31	Pramel7	no cluster
uc012clt.1	32	Sycp2	4
uc008ohn.1	33	Sycp2	4
uc009fgn.1	34	Crxos1	5
uc009fgo.2	35	Crxos1	5
uc012ezy.1	36	Crxos1	5
uc012ezz.1	37	Crxos1	5
uc011wiu.1	38	Gm4850	no cluster
uc009gac.1	39	Lgals6	6
uc009gad.1	40	Lgals6	6
uc011ypq.1	41	Gm1995	no cluster
uc011ypt.1	42	Gm7104	no cluster
uc008vqh.2	43	gm13057	7
uc008vqi.1	44	gm13057	7
uc012dol.1	45	gm13057	7
uc012dom.1	46	gm13057	7
uc012don.1	47	gm13057	7
uc012dor.1	48	gm13057	7
uc008kob.1	49	Pramel6	no cluster
uc009nkt.1	50	AK076897	no cluster
uc009nks.1	51	Hydin	no cluster
uc008wmo.2	52	Speer4d	no cluster
uc008wmr.1	53	Speer4d	8
uc012dsk.1	54	Speer4d	8
uc008wmn.1	55	Speer4d	8
uc007zjn.1	56	Dppa2	no cluster
uc007ljr.1	57	AK009035	9
uc007ljs.1	58	BC119473	9
uc007ljq.2	59	B1AQB2	no cluster
uc007ljp.2	60	Krtap4-13	no cluster
uc009hby.1	61	Nell1	no cluster
uc007lba.1	62	Gip	no cluster
uc009shv.2	63	tcstv1	no cluster
uc008unr.1	64	Rims3	10
uc008uns.1	65	Rims3	10
uc007nzw.1	66	Vti1b	11
uc007nzx.2	67	Vti1b	11
uc007nzy.2	68	Vti1b	11
uc007nzv.2	69	Arg2	no cluster
uc007grv.2	70	NM_011461	no cluster
uc011xli.1	71	Spic	no cluster
uc009itr.1	72	Dub2	12
uc009vep.1	73	Dub2	12
uc009itk.1	74	Dub2	12
uc009szr.2	75	Fam70a	13
uc009szs.2	76	Fam70a	13
uc008dtd.2	77	1110020A21Rik	14
uc012ayc.1	78	1110020A21Rik	14
uc012ayd.1	79	Pppm1b	no cluster
uc007hjr.1	80	Stac3	15
uc007hjs.1	81	Stac3	15
uc008rxe.1	82	Fam110b	16
uc008rxf.1	83	Fam110b	16
uc008rxg.1	84	Fam110b	16
uc009dui.1	85	Tuba3a	17
uc009erq.1	86	Tuba3a	17
uc009dug.1	87	4930417O13Rik	no cluster
uc008tjm.1	88	Ptprd	18
uc008tjo.1	89	Ptprd	18
uc008tjp.1	90	Ptprd	18
uc008xkd.2	91	Ppargc1a	19
uc008xkc.2	92	Ppargc1a	19
uc007npv.2	93	Clec14a	no cluster
uc007nxo.1	94	Syne2	20
uc007nxp.1	95	Syne2	20
uc007qnj.1	96	Ror2	no cluster
uc007odj.1	97	Rbm25	21
uc007odm.2	98	Rbm25	21
uc007uul.1	99	Pcdh9	22
uc011zpc.1	100	Pcdh9	22
